# DNA databases of an important tropical timber tree species *Shorea* *leprosula* (Dipterocarpaceae) for forensic timber identification

**DOI:** 10.1038/s41598-022-13697-x

**Published:** 2022-06-09

**Authors:** Chin Hong Ng, Kevin Kit Siong Ng, Soon Leong Lee, Nurul-Farhanah Zakaria, Chai Ting Lee, Lee Hong Tnah

**Affiliations:** Genetics Laboratory, Forest Research Institute Malaysia, 52109 Kepong, Selangor Malaysia

**Keywords:** Population genetics, Forestry

## Abstract

International timber trade communities are increasingly demanding that timber in the wood supply chain be sourced from sustainably harvested forests and certified plantations. This is to combat illegal logging activities to prevent further depletion of our precious forests worldwide. Hence, timber tracking tools are important to support law enforcement officials in ensuring only sustainably harvested timbers are traded in the market. In this study, we developed chloroplast DNA (cpDNA) and simple sequence repeat (SSR) databases as tracking tools for an important tropical timber tree species, *Shorea* *leprosula* from Peninsular Malaysia. A total of 1410 individual trees were sampled from 44 natural populations throughout Peninsular Malaysia. Four cpDNA regions were used to generate a cpDNA haplotype database, resulting in a haplotype map comprising 22 unique haplotypes derived from 28 informative intraspecific variable sites. This cpDNA database can be used to trace the origin of an unknown log at the regional level. Ten SSR loci were used to develop the SSR allele frequency database. Bayesian cluster analysis divided the 44 populations into two genetic clusters corresponding to Region A and Region B. Based on conservativeness evaluation of the SSR databases for individual identification, the coancestry coefficients (*θ*) were adjusted to 0.1900 and 0.1500 for Region A and B, respectively. These databases are useful tools to complement existing timber tracking systems in ensuring only legally sourced timbers are allowed to enter the wood supply chain.

## Introduction

*Shorea* *leprosula* Miq. (locally known as Meranti Tembaga) is a tropical tree species belonging to the Dipterocarpaceae family native to Southeast Asia, where it is widely distributed throughout the tropical rainforests of Peninsular Malaysia, Sumatra, and Borneo^1^. It is commonly found in lowland and hill dipterocarp forests below 700 m elevation^1^. It is classified as a near-threatened species under the International Union for Conservation of Nature (IUCN) Red List^2^. The harvested wood is internationally traded under the Light Red Meranti timber group as a general utility timber for the production of furniture, panelling, flooring, and plywood^3^. Due to easy accessibility of the species from lowland forests, it is prone to be targeted for illegal logging. Illegal logging is a lucrative business, which is associated with a total global market value between USD30-157 billion annually^4^. To address the issue of illegal logging, consumer countries have developed measures to ban the import of illegally-logged timber by implementing legality verification systems; such as through the United States Lacey Act (2008), the European Union Timber Regulation (2010), Australia Illegal Logging Prohibition Act (2012), the Japanese Clean Wood Act (2017); and most recently, the United Kingdom Timber Regulation (2021). In addition, the United Nations Office on Drugs and Crime (UNODC) has also produced a guide on best practices for forensic timber identification, aiming to overcome the challenges posed by illegal logging and environmental degradation ^5^. Such a complex set of urgent issues need to be addressed as reports have shown that an area of forest approximately equivalent to the size of Austria (83,871 km^2^) disappears worldwide every year as the result of illegal logging^6^.

To prevent illegal timber from entering the wood supply chain, enforcement authorities use timber tracking methods to verify the legality of the harvested wood. The most commonly used methods include paper based documentation, painted identification marks and radio frequency identification (RFID) tags^7^. In comparison, methods based on inherent wood characteristics such as visual identification (wood anatomy), genetics (DNA barcoding, haplotype map, DNA profiling), and chemical methods (stable isotopes, mass spectrometry and near infrared spectroscopy) can provide more reliable forensic timber identification^8^. Each tool has its own strength and in combination they complement one another allowing authorities to overcome limitations of more traditional methods in species identification, geographic origin verification or linking illegal logs to the stumps of origin.

Genetic approaches have been used to determine the origin of wood samples from many important species, including *Neobalanocarpus* *heimii*^9,10^, *Gonystylus* *bancanus*^11^, *Acer* *macrophyllum*^12^, *Cedrela* *odorata*^13^ and *Chamaecyparis* *taiwanensis*^14^. To develop timber tracking tools suitable for these species, researchers applied the principles of population genetics such as mutation, genetic drift, migration, adaptation, and speciation^8^. These methodologies utilise genetic material (genetic markers) common across groups of individuals to define populations for provenance testing or to define species for species identification^8^. During forensic timber identification, enforcement officers need to identify unknown samples at genus or species level correctly from the start, before further investigating geographic origin or individual identification. This process is commonly supported by wood anatomists through the examination of the internal structure of the specimen in comparison to reference materials^15^. In addition, such identifications can also be achieved using DNA barcoding technology based on nucleotide variation at specific gene regions^16^. One example of this is the CITES listed species with the genus *Gonystylus* which can be distinguished from other closely related species using a combination of genetic markers including internal transcribed spacer (ITS2), *trn*H-*psb*A intergenic spacer and *trn*L^11^. Once the particular species is identified, we can track the geographic region of origin using a population identification database developed from cpDNA^9,17^ or single nucleotide polymorphisms (SNP) markers^13^. If the suspected log can be traced back to a particular geographic region, we can use an individual identification database to link the log to the original stump^11,12^. Subsequently, the confidence level of the match probability can be tested by calculation of random match probability between the log and stump.

The Forest Research Institute Malaysia (FRIM) has developed comprehensive DNA profiling databases for several important tropical timber species for timber tracking, namely *N.* *heimii*^10^, *G.* *bancanus*^11^, *S.* *platyclados*^18^, *Intsia* *palembanica*^19^ and *Aquilaria* *malaccensis*^20^. As an extension, this study aimed to develop tracking tools for *S.* *leprosula* in the context of forensic identification. Specifically, we utilised cpDNA markers to develop a haplotype database and SSR markers to establish an allele frequency database for this important tropical timber species. We can use the cpDNA haplotype database to infer the geographic origin of an unknown sample to the regional level. Subsequently, by using the SSR allele frequency database, we can calculate the random match probability to support the strength of evidence in cases where the suspect log matches the tree stump. This gives a new impetus for higher acceptance of evidence by the judge, which will improve the success rate of prosecutions of illegal logging perpetrators.

## Results

### cpDNA haplotype database

**Figure 1:**
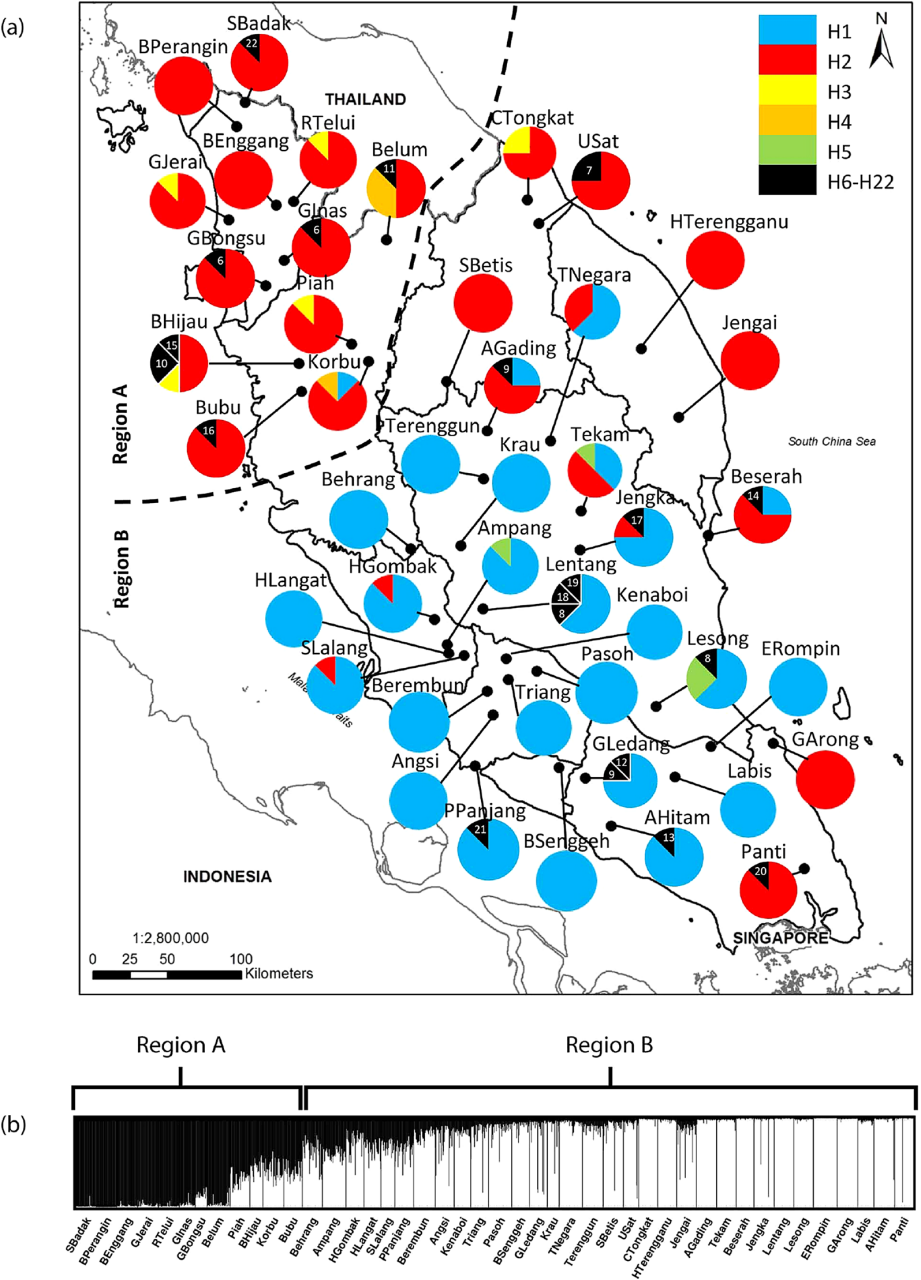
(**a**) Chloroplast haplotype distribution in the *Shorea* *leprosula* populations. The pie chart colours indicate haplotype distributions; and sector areas are proportional to sample size (Map was generated by ArcGIS-ArcMap version 10.8). (**b**) STRUCTURE analysis identified two clusters (*K* = 2) corresponding to Region A and B.

DNA sequencing of the choloroplast (cp) markers produced sequences of the following lengths: 573 bp (*atp*B-*rbc*L); 487 bp (*pet*G-*trn*P); 500 bp (*trn*L1-*trn*L2); and 593 bp (*psb*M-*trn*D). Alignment of the 352 individuals from the 44 populations yielded a total 28 variable sites: 11 in the *atp*B-*rbc*L spacer, seven in both the *pet*G-*trn*P and *psb*M-*trn*D spacers, and three in the *trn*L1-*trn*L2 spacer (Supplementary Table S1). Based on these 28 variable sites (21 base substitutions and 7 deletions) across the combined intergenic regions, a total of 22 unique haplotypes were found (Fig. 1a).

### SSR allele frequency database

**Figure 2:**
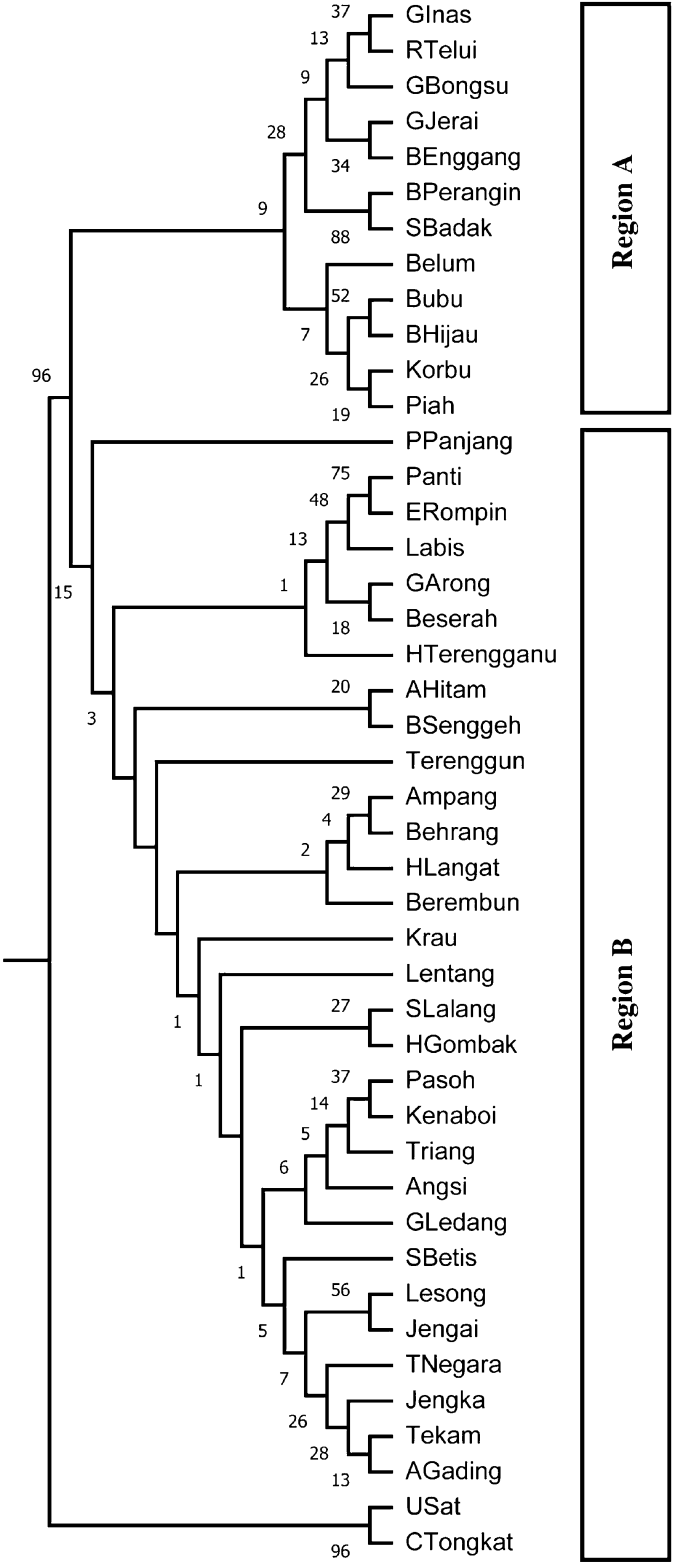
Dendrogram showing the relationship between 44 populations of *Shorea* *leprosula* in Peninsular Malaysia based on the UPGMA cluster analysis of SSR markers.

The reproducibility of SSR genotyping was confirmed by achieving consistent genotypes from five independent PCR amplifications on a single individual for each of the ten SSR loci. Individual bar plots from STRUCTURE analysis are presented in Fig. 1b. At the highest Delta *K* likelihood scores, the best representation of the data was *K* = 2 suggesting that the 44 populations in Peninsular Malaysia can be divided into two main genetic clusters: Region A and Region B. The first cluster, ‘Region A’ consists of 12 populations, namely SBadak, BPerangin, BEnggang, GJerai, RTelui, GInas, GBongsu, Belum, Piah, BHijau, Korbu and Bubu. The second cluster, ‘Region B’ consists of 32 populations, namely Behrang, Ampang, HGombak, HLangat, SLalang, PPanjang, Berembun, Angsi, Kenaboi, Triang, Pasoh, BSenggeh, GLedang, Krau, TNegara, Terenggun, SBetis, USat, CTongkat, HTerengganu, Jengai, AGading, Tekam, Beserah, Jengka, Lentang, Lesong, ERompin, GArong, Labis, AHitam and Panti. Similarly, the UPGMA dendrogram analysis also divided the 44 populations into two genetic clusters (Fig. 2) corresponding to Region A and B of the STRUCTURE result.

SSR allele frequency databases were established according to Region A and B, and characterized to evaluate the relative usefulness of each SSR marker in forensic investigation. The distribution of allele frequencies for each locus is listed in Table S2 (Region A database) and Table S3 (Region B database). Forensic parameters are shown in Table 1, with a total of 143 alleles and 174 alleles detected in the Region A and B databases, respectively. The observed (*H*_o_) and expected (*H*_e_) heterozygosity ranged from 0.3570 to 0.8346 and 0.4375 to 0.8795, respectively for populations in the Region A database; and ranged from 0.3298 to 0.8356 and 0.3469 to 0.8793, respectively for populations in the Region B database. The power of discrimination (PD) for the SSR loci ranged from 0.601 to 0.972 and 0.554 to 0.975, in Region A and B databases, respectively. The most discriminating locus was *Sle*605 in both the Region A (PD = 0.972) and Region B (PD = 0.975) databases. Minimum allele frequency was adjusted for alleles falling below the thresholds of 0.0066 (Region A) and 0.0024 (Region B).

**Table 1:** Genetic diversity and forensic variables (*A*: total number of alleles; *H*_o_: observed heterozygosity; *H*_e_: expected heterozygosity; PIC: polymorphic information content; HWE: Hardy–Weinberg equilibrium; MP: matching probability; PD: power of discrimination) for each the 10 SSR loci of *Shorea* *leprosula* in the Region A and B databases.

	*Sle*T01	*Sle*T09	*Sle*T11	*Sle*T15	*Sle*T17	*Sle*T29	*Sle*T31	*Sle*267	*Sle*465	*Sle*605
**Region A**										
*A*	19	13	12	11	7	16	8	13	16	28
*H*_*o*_	0.8346	0.7769	0.4211	0.5197	0.3570	0.6903	0.6011	0.5289	0.6913	0.7763
*H*_*e*_	0.8702	0.8063	0.4864	0.5763	0.4375	0.7627	0.6356	0.5631	0.7856	0.8795
PIC	0.8600	0.7800	0.4600	0.5100	0.3700	0.7300	0.5900	0.5300	0.7600	0.8700
HWE	0.0075	0.0328	0.0002*	0.0000*	0.0011*	0.0073	0.0065	0.1399	0.0013*	0.0117
MP	0.0320	0.0660	0.3180	0.2530	0.3990	0.0910	0.1860	0.2270	0.0730	0.0280
PD	0.9680	0.9340	0.6820	0.7470	0.6010	0.9090	0.8140	0.7730	0.9270	0.9720
**Region B**										
*A*	23	15	14	14	8	23	15	15	16	31
*H*_*o*_	0.8260	0.7872	0.3298	0.4674	0.4689	0.7237	0.6819	0.6667	0.7118	0.8356
*H*_*e*_	0.8658	0.8312	0.3469	0.4925	0.5534	0.7623	0.7240	0.7027	0.8043	0.8793
PIC	0.8500	0.8100	0.3300	0.4400	0.4500	0.7300	0.6900	0.6800	0.7800	0.8700
HWE	0.0014*	0.2187	0.0047*	0.0000*	0.0028*	0.0029*	0.0029*	0.3099	0.0109	0.2766
MP	0.0320	0.0490	0.4460	0.3120	0.2810	0.0870	0.1110	0.1200	0.0620	0.0250
PD	0.9680	0.9510	0.5540	0.6880	0.7190	0.9130	0.8890	0.8800	0.9380	0.9750

*Significant deviations from HWE after Bonferroni adjustment (*P* < 0.05/10 = 0.0050).

Deviations from HWE were detected in four of the SSR loci for Region A (*Sle*T11, *Sle*T15, *Sle*T17 and *Sle*465) and six SSR loci in Region B (*Sle*T01, *Sle*T11, *Sle*T15, *Sle*T17, *Sle*T29 and *Sle*T31). We evaluated these loci in each population independently to rule out the possible presence of null alleles. There were four populations in Region A (GJerai, RTelui, GBongsu and Piah) where a single one locus deviated from HWE; whereas there were eight populations in Region B (Behrang, HGombak, SLalang, Angsi, Klau, USat, Jengka and Panti) with a single locus and a single population (GLedang) with two loci that deviated from HWE (Table S4). Observed deviation from HWE was substantially lower in each population (either absence or not more than two loci) and thus it might be due to Wahlund effect caused by population substructuring in both Region A and B. Linkage disequilibrium (LD) testing was used to evaluate the independence of frequencies for all the SSR genotypes. A total of 13.3% and 28.9% of the 45 pairwise loci were found significant evidence of LD for Region A and B, respectively. Some of the loci might be linked as a result of population substructuring and inbreeding (inbreeding coefficient = 0.0822 [Peninsular Malaysia]). These results are in line with observations in real populations, where the assumption of completely random mating and zero migration required for HWE and LD are unlikely to be met, either in humans, animals or plants ^21–23^.

Mean self-assignment, the proportion of individuals correctly assigned back to their population, was 45.9% and ranged from 14.3% (Kenaboi) to 81.3% (CTongkat) between population (Table 2). At the regional level, correct assignment rate of individuals to their region of origin was higher, 87.4% for Region A and 90.0% for Region B, (average of 88.7%).

**Table 2:** Self-assignment test outcomes for *Shorea* *leprosula* individuals at the population and regional levels.

Population	Correctly assigned (%)	Region	Correctly assigned (%)
SBadak	70.6	Region A	87.4
BPerangin	73.6		
BEnggang	65.7		
GJerai	62.9		
RTelui	57.1		
Ginas	44.4		
GBongsu	30.0		
Belum	51.3		
Piah	45.5		
BHijau	50.0		
Korbu	48.6		
Bubu	38.7		
Behrang	52.9	Region B	90.0
Ampang	50.0		
HGombak	50.0		
HLangat	41.4		
Slalang	39.1		
PPanjang	46.9		
Berembun	55.6		
Angsi	34.4		
Kenaboi	14.3		
Triang	33.3		
Pasoh	25.6		
BSenggeh	53.3		
GLedang	26.7		
Krau	20.0		
TNegara	30.8		
Terenggun	60.0		
SBetis	47.1		
Usat	46.2		
CTongkat	81.3		
HTerengganu	37.5		
Jengai	18.2		
AGading	32.4		
Tekam	33.3		
Beserah	53.3		
Jengka	35.3		
Lentang	63.6		
Lesong	27.3		
Erompin	65.0		
GArong	65.7		
Labis	33.3		
AHitam	57.1		
Panti	48.4		
Mean	45.9	Mean	88.7

### Conservativeness of the database

The coancestry coefficient (*θ*) for Peninsular Malaysia (0.0579) was higher than those of Region A (0.0454) and Region B (0.0500) (Table 3). A total of 4.54% and 5.00% of the genetic variability was distributed among populations within Region A and Region B, respectively. In terms of inbreeding coefficient (*f*), the value for the Region A database (*f* = 0.0892) was highest, followed by Peninsular Malaysia (*f* = 0.0822) and Region B (*f* = 0.0666). All the *θ* and *f* values were significantly greater than zero, demonstrated by the 95% confidence intervals not overlapping with zero. Both of the *θ* and *f* values were used to calculate the conservativeness of each database by testing the cognate database (*P*_origin_) against the regional database (*P*_combined_). The databases were non-conservative at the calculated *θ* value. In order for both the Region databases (A and B) to be conservative, the value of *θ* was adjusted from 0.0454 to 0.1900 for Region A and from 0.0500 to 0.1500 for Region B. For the Region A database, the most common SSR profile frequency is 2.69 × 10^–7^ or 1 in 3.72 million and the rarest profile frequency is 1.84 × 10^–14^ or 1 in 54.3 trillion. For the Region B database, the most common SSR profile frequency is 1.06 × 10^–7^ or 1 in 9.43 million and the rarest profile frequency is 4.03 × 10^–16^ or 1 in 2.48 quadrillion.

**Table 3:** Coancestry (*θ*) and inbreeding (*f*) coefficients for *Shorea* *leprosula* at each hierarchical level.

Hierarchical level	Coancestry coefficient (*θ*)			Inbreeding coefficient (*f*)		
	Mean	2.5%	97.5%	Mean	2.5%	97.5%
Peninsular Malaysia (N = 1410)	0.0579	0.0475	0.0741	0.0822	0.0630	0.1075
Region A (N = 381)	0.0454	0.0389	0.0528	0.0892	0.0662	0.1151
Region B (N = 1029)	0.0500	0.0399	0.0658	0.0666	0.0500	0.0871

## Discussion

At the moment, the database is not accessible to the public. However, the public can contribute by reporting suspicious illegal logging activities to the relevant enforcement unit, so that actions can be taken and suspicious samples collected for forensic timber identification. In addressing forensic timber identification questions, we use a cpDNA haplotype database to investigate the geographic origin of a suspect log at a regional level. Subsequently, we use an SSR allele frequency database to narrow down the geographic origin to population level. After the population is identified, the state forest department’s enforcement officials can verify in their system if the area was permitted for logging activities. If that area is a forest reserve where no logging permit is being issued, efforts to locate potential stumps belonging to the sampled log can be initiated. Once potential stumps are found, we can try to link the log to the potential stumps by comparing their SSR DNA profiles. A random match probability between the log and the potential stumps can be established by using the SSR allele frequency database.

From the cpDNA haplotype database, haplotypes H1 and H2 were most prevalent in Peninsular Malaysia, with a frequency of 47.2% and 42.6%, respectively. The distribution of cpDNA haplotypes is overlaid by the division of populations in Region A and B as suggested by the STRUCTURE analysis. For Region A, haplotype H2 was found in all the populations, either in all the samples (BPerangin and BEnggang) or part of the samples. The less common haplotypes, H3 (1.7%) and H4 (1.1%) were also found in Region A. Overall, we observed haplotype H2 dominates the populations in this region. Whereas for Region B, 78% of populations carried haplotype H1 in all the samples (Terenggun, Krau, Behrang, HLangat, Berembun, Kenaboi, Angsi, Triang, Pasoh, BSenggeh, Labis and ERompin), with the exception of some populations which exhibited part of their samples carried haplotype H1 (TNegara, AGading, Tekam, Jengka, Beserah, Ampang, Lentang, HGombak, SLalang, Lesong, GLedang, PPanjang and AHitam). In addition, the less common haplotype H5 (1.1%) is found solely in this region. As a whole, haplotype H1 dominates the populations in Region B. Those haplotype H2 found in the populations of Region B might be due to the retention of ancestral polymorphism by the maternally inherited cpDNA marker^24^. The remaining rare haplotypes, H6-H22, present in one or two individuals are endemic to certain populations, as shown in Fig. 1a.

Based on the cpDNA haplotypes, *S.* *leprosula* individuals from Peninsular Malaysia can be traced back to their geographical origin in either Region A or B. In forensic investigation, if the generated haplotype of an unknown log belongs to haplotype H3 or H4, we can postulate that it might have originated from Region A. Similarly, if haplotype H1 or H5 were detected, then Region B would be the most likely source of origin. However, based only on the cpDNA haplotype database, it is impossible to track an unknown log back to a specific population or forest reserve because forest reserve boundaries were defined according to political governance and thus may not necessarily reflect the distribution of natural populations of the species. It should be noted that some rare haplotypes might not be represented in the database, as it is impossible to collect all *S.* *leprosula* trees from every forest reserve in Peninsular Malaysia. We can include more sampling sites in the future to improve the comprehensiveness of the cpDNA haplotype database. Particularly, the inclusion of populations from other distributions such as Sumatra and Borneo could provide some insights on the evolutionary history and gene flow of the species due to isolation and separation by South China Sea between Peninsular Malaysia and Borneo as well as by Straits of Malacca between Peninsular Malaysia and Sumatra.

Once the geographical origin at regional level is ascertained, an assignment test based on the SSR allele frequency database can be used to trace the samples origin to population level. In this study, we observed low assignment rates to origin populations, which may be due to the weak genetic structure (*θ* = 0.058) observed in this species. The value of *θ* shows that only 5.8% of genetic variability was found distributed among populations, thus suggesting high genetic similarity. This *θ* value (0.058) was higher than *I.* *palembanica* (0.026)^19^ and *S.* *platyclados* (0.033)^18^ but lower than *G.* *bancanus* (0.067)^11^, *A.* *malaccensis* (0.097)^20^ and *N.* *heimii* (0.127)^10^. Previous study suggested that populations of *S.* *leprosula* sampled from Peninsular Malaysia were a continuous, connected forest in the past, particularly in the low inland forests^25^. Continuous distribution would promote gene flow among populations through the sharing of a common gene pool, as shown by the common haplotypes H1 and H2 observed in the cpDNA population database. The current mean assignment rate at the population level is 45.90%, which is lower than those seen in other tropical species such as *G.* *bancanus* (54.80%)^11^, *I.* *palembanica* (62.20%)^19^, *S.* *platyclados* (77.78%)^18^ and *A.* *malaccensis* (92.09%)^20^. At the regional level, the mean assignment rate to region is 88.70%, which is higher than seen in *I.* *palembanica* (80.21%)^19^ but lower than *A.* *malaccensis* (94.96%)^20^, *S.* *platyclados* (99.11%)^18^ and *G.* *bancanus* (100%)^11^.

The identification of illegal logging sites can be achieved under two circumstances. Firstly, by utilising assignment tests based on the SSR allele frequency database to locate the original population for the suspected illegal log. Secondly, if the Forest Department has received report on illegal logging activities in a specified area. As such, with help from experienced foresters and local indigenous people who are familiar with their local forest area, it is possible to find and sample the potential stumps which potentially match the suspect log within the forest. Once potential stumps are found, a tissue sample can be collected for DNA testing following FRIM’s standard operating protocol on DNA forensics for plant species identification and wood tracking^26^. If the suspect log shows a similar SSR profile to a particular stump, we can calculate a random match probability by using the SSR allele frequency database with corrected *θ* value. By considering both population substructuring and inbreeding coefficient, the adjusted *θ* value will increase the profile frequency but conversely, understating the weight of the DNA evidence against a defendant^27^, should the matter be brought before the legal system. Random match probability is the reciprocal of profile frequency (1/profile frequency), representing the estimated frequency at which a particular SSR profile would be expected to occur in a population^21^. This will help to determine the probability of a match between an unknown log and its potential origin stump. The possible profile frequency based on the 10 SSR loci ranges between the profile frequency of the most common genotype which would be the least powerful in terms of differentiating between two unrelated individuals ^28^, and the rarest theoretical profile. Based on the Region A database, the possible SSR profile frequencies range from 2.69 × 10^–7^ to 1.84 × 10^–14^, and for the Region B database, from 1.06 × 10^–7^ to 4.03 × 10^–16^. With such low profile frequencies, we can rule out the possibility of a random match between the DNA profiles of any log and stump^21^.

In this study, we obtained cpDNA fragment and SSR loci using high quality samples such as inner bark or leaf tissue preserved in liquid nitrogen. However, many seized woods or logs are usually have been dried or processed in practice. Thus, this may pose a challenge to extract sufficient and good quality DNA from dry wood for subsequent DNA analysis. To close the DNA extraction gap in *S.* *leprosula*, our future study is to develop a suitable DNA extraction method for dry wood and processed sample. The extracted DNA is then tested by PCR amplification on both cpDNA and SSR markers utilized in the DNA databases.

## Conclusions

We report on the development of cpDNA haplotype and SSR allele frequency databases for an important timber species, *S.* *leprosula* in Peninsular Malaysia. The cpDNA haplotype database enables the tracing of unknown log at the regional level. The SSR allele frequency database was validated for specificity and accuracy for the calculation of random match probability of an unknown log to a potential origin stump. This database along with the existing reference databases in other important forest timber species will serve as an impetus and increase the use of DNA technology in illegal logging investigations and verification of legality in wood supply chains.

## Methods

### Sample collection and DNA extraction

In this study, 1,410 *S.* *leprosula* wild samples representing 44 populations from the natural forests distributed throughout Peninsular Malaysia (Table 4) were collected. The sample collection was carried out with the permissions granted from the State Forest Departments (Kedah, Perak, Kelantan, Terengganu, Pahang, Selangor, Negeri Sembilan, Melaka and Johor), the Department of Wildlife and National Parks, Royal Belum State Park and Johor National Parks Corporation. The voucher specimen was identified by Ramli Ponyoh and deposited in FRIM herbarium centre (voucher number = A4363). Cambium or leaf tissues was collected from each sample and kept in liquid nitrogen during transportation from the field to laboratory. Total genomic DNA was extracted using the 2× cetyltrimethylammonium bromide (CTAB)^29^ procedure and purified using the High Pure PCR Template Preparation Kit (Roche Diagnostics, GmbH, Penzberg, Germany). For cpDNA analysis, eight purified DNA samples per population were used (8 × 44 populations = 352 samples), while all the purified DNA samples (1,410 samples) were used for SSR analysis.

**Table 4:** Names, geographic locations, altitude and sample number for 44 populations of *Shorea* *leprosula* in Peninsular Malaysia.

Population	Acronym	Latitude (N)	Longitude (E)	Altitude/m	Number of samples
Sungai Badak	SBadak	6.47	100.54	240	34
Bukit Perangin	BPerangin	6.32	100.49	133	35
Bukit Enggang	BEnggang	5.84	100.73	282	35
Gunung Jerai	GJerai	5.75	100.44	138	35
Rimba Telui	RTelui	5.86	100.84	166	35
Gunung Inas	GInas	5.50	100.78	105	27
Gunung Bongsu	GBongsu	5.35	100.67	206	20
Belum	Belum	5.63	101.40	275	39
Piah	Piah	4.99	101.19	110	33
Bintang Hijau	BHijau	4.87	100.87	550	22
Korbu	Korbu	4.89	101.29	616	35
Bubu	Bubu	4.70	100.89	289	31
Behrang	Behrang	3.74	101.56	440	34
Ampang	Ampang	3.16	101.78	55	40
Hulu Gombak	HGombak	3.31	101.70	158	30
Hulu Langat	HLangat	3.10	101.79	343	29
Sungai Lalang	SLalang	3.09	101.88	65	23
Pasir Panjang	PPanjang	2.42	101.95	47	32
Berembun	Berembun	2.87	102.02	410	36
Angsi	Angsi	2.73	102.06	460	32
Kenaboi	Kenaboi	3.07	102.14	458	28
Triang	Triang	2.94	102.15	202	30
Pasoh	Pasoh	2.99	102.32	140	39
Bukit Senggeh	BSenggeh	2.40	102.46	98	30
Gunung Ledang	GLedang	2.34	102.62	107	30
Krau	Krau	3.76	101.86	77	20
Taman Negara	TNegara	4.40	102.40	105	39
Terenggun	Terenggun	4.17	102.00	139	35
Sungai Betis	SBetis	4.76	101.77	223	34
Ulu Sat	USat	5.73	102.33	68	26
Chabang Tongkat	CTongkat	5.88	102.26	88	32
Hulu Terengganu	HTerengganu	4.97	102.95	57	32
Jengai	Jengai	4.55	103.18	84	33
Aur Gading	AGading	4.46	102.02	259	34
Tekam	Tekam	3.97	102.59	78	33
Beserah	Beserah	3.83	103.36	194	30
Jengka	Jengka	3.74	102.58	93	34
Lentang	Lentang	3.38	101.99	124	33
Lesong	Lesong	2.78	103.04	92	33
Endau Rompin	ERompin	2.53	103.38	48	40
Gunung Arong	GArong	2.55	103.76	30	35
Labis	Labis	2.35	103.16	65	27
Ayer Hitam	AHitam	2.05	102.77	25	35
Panti	Panti	1.79	103.94	44	31

### cpDNA haplotype database

The target cpDNA regions were amplified using four intergenic spacer primers namely *atp*B-*rbc*L, *pet*G-*trn*P, *trn*L1-*trn*L2 and *psb*M-*trn*D (Supplementary Table S5). These four cpDNA markers were selected based on their informative intraspecific variability identified in eight individuals from different populations of *S.* *leprosula*. Each reaction was performed in a 10 μL total volume composed of 1× Type-it Multiplex PCR Master Mix (Qiagen), 0.2 μM each primer and 10 ng of template DNA on a 2720 Thermal Cycler (Applied Biosystems, Foster City, CA). The thermal cycling conditions consist of a first activation step at 95 °C for 5 min, followed by 35 cycles of denaturation at 95 °C for 30 s, annealing at 50 °C for 90 s, and extension at 72 °C for 1 min; with a final extension step at 60 °C for 30 min. An ABI 3130xl capillary sequencer (Applied Biosystems) was used to sequence both forward and reverse directions of each PCR product. The sequence data was edited and analysed using Sequencher v.5.1 (Gene Codes Corporation, Michigan, USA). We identified chloroplast haplotypes by taking into account insertion/deletion, and substitution among sequences. A total of 32 sequences were deposited in GenBank with accession numbers ranging from MZ419000 to MZ419031.

### SSR allele frequency database

We used ten SSR loci, namely *Sle*T01, *Sle*T09, *Sle*T11, *Sle*T15, *Sle*T17, *Sle*T29, *Sle*T31 (unique to this study: Supplementary material S1), *Sle*267, *Sle*465 and *Sle*605^30^ to genotype the 1410 individuals. Information on primer sequence, repeat motifs, allele size range, fluorescent label and GenBank accession number for the ten SSR markers are listed in supplementary Table S6. The PCR mix consists of 1× Type-it Multiplex PCR Master Mix (Qiagen), 0.4 μM for each primer and 10 ng of template DNA. The forward primer was fluorescently labelled and mixed with nonlabelled primer at a ratio of 1:10. PCR amplification was performed using the programme: activation step at 95 °C for 5 min, followed by 40 cycles of a denaturation step at 95 °C for 30 s, annealing at 55 °C for 90 s, and extension at 72 °C for 30 s; and a final extension at 72 °C for 45 min. PCR products were electrophoretically separated using an ABI 3130xl capillary sequencer (Applied Biosystems) with GeneScan 400HD ROX size standard (Applied Biosystems). Fragment sizes were determined using GeneMarker v2.6.4 software (Soft Genetics LLC, Pennsylvania, USA). To evaluate the reproducibility of all SSR loci, five independent PCR amplifications were performed on one individual^31^.

### Statistical analysis 

For cluster analysis, we used STRUCTURE v2.3.4 to run our dataset using a burn-in length of 100,000 and 200,000 steps for the Markov Chain Monte Carlo (MCMC)^32^. We applied models of admixture with sampling locations included as prior population information. Correlated allele frequencies were applied with *K* values ranging from 1 to 10 for 10 repetitions. The optimal number of genetic clusters was estimated based on the Delta *K* method^33^ of STRUCTURE SELECTOR^34^. For the optimal *K*, data from the 10 independent runs of STRUCTURE analyses were graphically represented using CLUMPAK^35^. To support the analysis of the population structure, a UPGMA dendrogram was constructed based on Nei’s *D*_A_ using POPTREE2^36^. 1000 bootstrap replicates were applied to determine the relative strength of the nodes.

The populations of *S.* *leprosula* were divided into two regions, Region A and Region B based on the optimal value of *K* = 2 derived above. Subsequently, the SSR database was built for Region A and B, comprising 381 (12 populations) and 1029 (32 populations) individuals, respectively. Allele frequency for each locus was calculated using Microsatellite Toolkit^37^. The number of alleles per locus (*A*), observed (*H*_o_) and expected heterozygosity (*H*_e_), conformity to Hardy–Weinberg equilibrium (HWE) expectations and linkage disequilibrium (LD) between loci were assessed using Fisher’s exact tests in Genetic Data Analysis (GDA) v1.1 ^38^. The *p* value for departure from HWE and LD was adjusted by Bonferroni correction^39^. Forensic parameters including polymorphic information content (PIC), matching probability (MP) and power of discrimination (PD) were assessed using PowerStats v1.2^40^. Coancestry (*θ*) and inbreeding (*f*) coefficients for the combined database (Peninsular Malaysia) and regional database (Region A and B) were calculated with 1000 bootstrap replicates in GDA^41^. Self-assignment tests were used to evaluate the proportion of correctly assigned individuals to the designated population and region as implemented in GENECLASS2^42^.

The subpopulation-cum-inbreeding model was used to calculate the profile frequency by multiplying the frequency of each locus across all the loci^43^. The most common and rarest profile frequencies were calculated by considering an individual sample that is heterozygous at all loci possessing the two most common alleles and rarest alleles at each locus, respectively. The conservativeness of the database was estimated by calculating the full profile frequency of each individual using genotype frequencies derived from the cognate database (*P*_origin_) against profile frequency of each individual using genotype frequencies derived from the regional database (*P*_combined_). The relative difference between the databases (*d*) were defined as *d* = log_10_ (*P*_origin_/*P*_combined_). If the *d* value was negative, in the case of *P*_origin_ was less than *P*_combined_, it suggests that the database is conservative^27^. For a non-conservative database, in the case of positive *d* value, a series of *θ* adjustments were applied to recalculate *P*_combined_ until all samples present a negative *d* value.

### Plant collection declaration

We declare that all our experimental research and field sampling of plant material comply with local, national or international guidelines and legislation.

## Supplementary Information

Supplementary Information.

## Data Availability

Raw sequence information and SSR primer pairs have been deposited to NCBI; GenBank accession numbers are provided in Table S6.
