# Supplementary material for: DNA databases of an important tropical timber tree species Shorea leprosula (Dipterocarpaceae) for forensic timber identification
Source: Sci Rep. 2022 Jun 9;12:9546. doi: 10.1038/s41598-022-13697-x (PMC9184630; doi:10.1038/s41598-022-13697-x)
Supplement: Supplementary file 1 — Supplementary Information. [file 41598_2022_13697_MOESM1_ESM.pdf]

Haplotype	*atp*B-*rbc*L											*pet*G-*trn*P							*trn*L1-*trn*L2			*psb*M-*trn*D						
	59	268	317	380	386	397	419-420	422	437-438	440-441	443-444	81-82	84	85-86	136	155	321	341	183	194	326	157	321	452	485	538	546	557
H01	C	C	C	C	T	T	-	-	AA	TG	TC	-	T	-	C	A	T	T	G	G	C	A	G	C	A	G	T	A
H02	C	C	C	C	T	K	-	-	AA	TG	TC	-	T	-	C	A	T	T	G	G	C	A	G	C	A	G	T	A
H03	C	C	C	C	T	K	-	-	AA	TG	TC	-	T	-	C	A	T	T	G	G	C	A	G	C	A	C	T	A
H04	A	C	C	C	C	K	-	-	AA	TG	TC	-	T	-	C	A	C	T	A	G	C	T	G	C	A	G	G	A
H05	C	C	C	C	T	T	-	-	AA	TG	TC	CT	A	AT	C	A	T	T	G	G	C	A	G	C	A	G	T	A
H06	A	C	C	C	T	K	-	-	AA	TG	TC	-	T	-	C	A	C	T	A	G	C	T	G	C	A	G	G	A
H07	A	C	C	C	C	T	-	-	AA	TG	TC	-	T	-	C	A	C	T	A	G	C	T	G	C	A	G	G	A
H08	C	C	C	A	C	T	-	-	AA	TG	TC	-	T	-	C	A	C	T	G	G	C	A	G	C	A	G	G	C
H09	C	C	T	C	T	T	-	-	AA	TG	TC	-	T	-	C	A	T	T	G	G	C	A	G	C	A	G	T	A
H10	C	C	C	C	T	K	-	-	AA	TG	TC	-	T	-	C	A	T	T	G	G	C	A	T	C	A	G	T	A
H11	A	C	C	A	C	K	-	-	AA	TG	TC	-	T	-	C	A	C	T	A	G	C	T	G	C	A	G	G	A
H12	C	C	C	C	C	T	-	-	AA	TG	TC	-	T	-	A	A	C	T	G	G	C	A	G	C	A	G	G	A
H13	A	C	C	C	A	T	-	-	AA	TG	TC	-	T	-	C	A	C	T	A	G	C	T	G	C	A	G	G	A
H14	C	C	C	C	C	K	-	-	AA	TG	TC	-	T	-	A	A	C	T	G	T	C	A	G	C	A	G	G	A
H15	C	T	C	C	T	K	-	-	AA	TG	TC	-	T	-	C	A	T	T	G	G	C	A	G	C	A	G	T	A
H16	C	C	C	C	T	K	-	-	AA	TG	TC	-	T	-	C	G	T	T	G	G	C	A	G	C	A	G	T	A
H17	C	C	C	C	T	T	TT	A	AA	TG	TC	-	T	-	C	A	T	T	G	G	C	A	G	C	A	G	T	A
H18	C	C	C	C	T	T	-	-	-	-	-	-	T	-	C	A	T	T	G	G	C	A	G	C	A	G	T	A
H19	C	C	C	C	T	T	-	-	AA	TG	TC	-	T	-	C	A	T	T	G	G	C	A	G	C	T	G	T	A
H20	C	C	C	C	T	K	-	-	AA	TG	TC	-	T	-	C	A	T	G	G	G	C	A	G	C	A	G	T	A
H21	C	C	C	C	T	T	-	-	AA	TG	TC	-	T	-	C	A	T	T	G	G	G	A	G	C	A	G	T	A
H22	C	C	C	C	T	K	-	-	AA	TG	TC	-	T	-	C	A	T	T	G	G	C	A	G	A	A	G	T	A

Table S1. Variable sites due to base substitution or indels in the plastid regions (*atp*B-*rbc*L, *pet*G-*trn*P, *trn*L1-*trn*L2 and *psb*M-*trn*D) of *Shorea leprosula* in the cpDNA haplotype database. The symbol - denotes deletion.

Allele size - frequency									
*Sle*T01	*Sle*T09	*Sle*T11	*Sle*T15	*Sle*T17	*Sle*T29	*Sle*T31	*Sle*267	*Sle*465	*Sle*605
217 ~ 0.0276	216 ~ 0.0039	268 ~ 0.0737	98 ~ 0.0105	202 ~ 0.0092	153 ~ 0.0026	108 ~ 0.0289	109 ~ 0.0184	53 ~ 0.1148	115 ~ 0.0026
218 ~ 0.1063	220 ~ 0.0013	270 ~ 0.0013	102 ~ 0.3123	206 ~ 0.7008	156 ~ 0.0328	112 ~ 0.2454	111 ~ 0.0026	55 ~ 0.0119	119 ~ 0.0158
219 ~ 0.0039	221 ~ 0.0236	272 ~ 0.0737	103 ~ 0.0118	207 ~ 0.0131	159 ~ 0.0577	117 ~ 0.1168	113 ~ 0.0053	57 ~ 0.0251	123 ~ 0.0553
221 ~ 0.0026	225 ~ 0.2192	274 ~ 0.0026	106 ~ 0.0315	208 ~ 0.0013	161 ~ 0.3688	121 ~ 0.5367	115 ~ 0.0013	59 ~ 0.0066	125 ~ 0.2500
222 ~ 0.0131	229 ~ 0.1220	276 ~ 0.7013	107 ~ 0.0039	210 ~ 0.2677	164 ~ 0.2953	126 ~ 0.0459	117 ~ 0.0105	62 ~ 0.0277	127 ~ 0.1500
225 ~ 0.0932	233 ~ 0.2428	277 ~ 0.0026	108 ~ 0.0131	214 ~ 0.0066	165 ~ 0.0171	130 ~ 0.0052	119 ~ 0.1000	64 ~ 0.3496	129 ~ 0.0566
226 ~ 0.1785	237 ~ 0.2559	278 ~ 0.0105	109 ~ 0.0302	218 ~ 0.0013	168 ~ 0.0669	146 ~ 0.0171	121 ~ 0.6316	66 ~ 0.2586	131 ~ 0.1263
229 ~ 0.1129	242 ~ 0.0630	280 ~ 0.1053	110 ~ 0.5696		169 ~ 0.0656	147 ~ 0.0039	123 ~ 0.1618	67 ~ 0.0040	133 ~ 0.0447
230 ~ 0.2310	246 ~ 0.0525	282 ~ 0.0053	112 ~ 0.0026		172 ~ 0.0210		125 ~ 0.0408	68 ~ 0.0910	135 ~ 0.0658
231 ~ 0.0092	250 ~ 0.0092	284 ~ 0.0158	114 ~ 0.0039		173 ~ 0.0118		127 ~ 0.0197	70 ~ 0.0409	137 ~ 0.0395
233 ~ 0.0092	254 ~ 0.0013	289 ~ 0.0066	117 ~ 0.0105		176 ~ 0.0262		129 ~ 0.0026	73 ~ 0.0303	139 ~ 0.0211
234 ~ 0.0853	258 ~ 0.0013	293 ~ 0.0013			177 ~ 0.0013		131 ~ 0.0013	75 ~ 0.0224	141 ~ 0.0513
237 ~ 0.0066	297 ~ 0.0039				180 ~ 0.0105		139 ~ 0.0039	77 ~ 0.0013	143 ~ 0.0526
238 ~ 0.0564					184 ~ 0.0066			79 ~ 0.0013	145 ~ 0.0066
242 ~ 0.0184					191 ~ 0.0013			81 ~ 0.0119	147 ~ 0.0013
245 ~ 0.0315					192 ~ 0.0144			83 ~ 0.0026	149 ~ 0.0079
246 ~ 0.0066									153 ~ 0.0039
249 ~ 0.0013									155 ~ 0.0092
250 ~ 0.0066									157 ~ 0.0039
									159 ~ 0.0066
									161 ~ 0.0053
									167 ~ 0.0013
									171 ~ 0.0013
									173 ~ 0.0079
									175 ~ 0.0039
									177 ~ 0.0013
									179 ~ 0.0053
									181 ~ 0.0026

Table S2. Allele frequencies for each of the 10 SSR markers of *Shorea leprosula* in the Region A database. The first number represents the allele size and second number represents the frequency. Minimum allele frequencies were adjusted for alleles falling below the threshold (5/2n = 0.0066).

Allele size - frequency									
*Sle*T01	*Sle*T09	*Sle*T11	*Sle*T15	*Sle*T17	*Sle*T29	*Sle*T31	*Sle*267	*Sle*465	*Sle*605
217 ~ 0.0044	216 ~ 0.0010	268 ~ 0.0302	98 ~ 0.0078	202 ~ 0.0107	151 ~ 0.0175	103 ~ 0.0049	103 ~ 0.0044	53 ~ 0.0127	115 ~ 0.0005
218 ~ 0.0243	220 ~ 0.0155	272 ~ 0.0447	100 ~ 0.0005	205 ~ 0.0005	153 ~ 0.0044	108 ~ 0.0238	107 ~ 0.0073	55 ~ 0.0093	119 ~ 0.0063
219 ~ 0.0029	221 ~ 0.0262	275 ~ 0.0049	102 ~ 0.2206	206 ~ 0.4523	156 ~ 0.1080	112 ~ 0.0637	111 ~ 0.0029	57 ~ 0.0039	121 ~ 0.0010
221 ~ 0.0005	225 ~ 0.2478	276 ~ 0.8016	103 ~ 0.0019	209 ~ 0.0350	159 ~ 0.0015	117 ~ 0.2082	113 ~ 0.0010	59 ~ 0.0054	123 ~ 0.0676
222 ~ 0.0471	229 ~ 0.1288	277 ~ 0.0005	106 ~ 0.0199	210 ~ 0.4908	161 ~ 0.376	121 ~ 0.4528	115 ~ 0.0287	62 ~ 0.0341	125 ~ 0.2549
225 ~ 0.1166	233 ~ 0.2046	278 ~ 0.0049	107 ~ 0.0010	214 ~ 0.0092	164 ~ 0.2782	122 ~ 0.0131	117 ~ 0.001	64 ~ 0.2945	127 ~ 0.0778
226 ~ 0.2206	237 ~ 0.0991	280 ~ 0.0871	108 ~ 0.0068	218 ~ 0.0010	165 ~ 0.0477	126 ~ 0.1435	119 ~ 0.0423	66 ~ 0.2405	129 ~ 0.0603
227 ~ 0.0015	242 ~ 0.0739	282 ~ 0.0005	109 ~ 0.0544	231 ~ 0.0005	167 ~ 0.0024	130 ~ 0.0438	121 ~ 0.4966	67 ~ 0.0010	131 ~ 0.0910
228 ~ 0.0005	245 ~ 0.0015	284 ~ 0.0068	110 ~ 0.6749		168 ~ 0.0452	132 ~ 0.0005	123 ~ 0.1249	68 ~ 0.1870	133 ~ 0.0798
229 ~ 0.1501	246 ~ 0.1812	297 ~ 0.0024	112 ~ 0.0010		169 ~ 0.0340	134 ~ 0.0146	125 ~ 0.0496	70 ~ 0.0151	135 ~ 0.0428
230 ~ 0.1696	250 ~ 0.0170	301 ~ 0.0005	113 ~ 0.0005		172 ~ 0.0229	139 ~ 0.0195	127 ~ 0.1545	73 ~ 0.1047	137 ~ 0.0520
231 ~ 0.0024	254 ~ 0.0010	305 ~ 0.0005	114 ~ 0.0097		173 ~ 0.0054	143 ~ 0.0015	129 ~ 0.0797	75 ~ 0.0560	139 ~ 0.0282
233 ~ 0.019	262 ~ 0.0015	306 ~ 0.0151	117 ~ 0.0005		176 ~ 0.0350	146 ~ 0.0088	131 ~ 0.0015	77 ~ 0.0268	141 ~ 0.0248
234 ~ 0.119	279 ~ 0.0005	308 ~ 0.0005	121 ~ 0.0005		177 ~ 0.0029	147 ~ 0.0010	133 ~ 0.0039	79 ~ 0.0068	143 ~ 0.1415
235 ~ 0.0063	297 ~ 0.0005				180 ~ 0.0039	151 ~ 0.0005	141 ~ 0.0019	81 ~ 0.0015	145 ~ 0.0302
237 ~ 0.0068					182 ~ 0.0029			83 ~ 0.0010	147 ~ 0.0088
238 ~ 0.0486					183 ~ 0.0005				149 ~ 0.0005
239 ~ 0.0010					184 ~ 0.0005				151 ~ 0.0039
241 ~ 0.0180					187 ~ 0.0010				153 ~ 0.0015
242 ~ 0.0292					188 ~ 0.0039				155 ~ 0.0005
246 ~ 0.0068					190 ~ 0.0005				159 ~ 0.0005
250 ~ 0.0039					191 ~ 0.0054				161 ~ 0.0029
254 ~ 0.0010					192 ~ 0.0005				163 ~ 0.0010
									165 ~ 0.0146
									167 ~ 0.0005
									169 ~ 0.0010
									173 ~ 0.0005
									175 ~ 0.0015
									177 ~ 0.0015
									179 ~ 0.0005
									183 ~ 0.0019

Table S3. Allele frequencies for each of the 10 SSR markers of *Shorea leprosula* in the Region B database. The first number represents the allele size and second number represents the frequency. Minimum allele frequencies were adjusted for alleles falling below the threshold (5/2n = 0.0024).

Region	Population	Significant deviations from HWE after Bonferroni adjustment ($P < 0.005$)
A	SBadak	nil
	BPerangin	nil
	BEnggang	nil
	GJerai	*Sle*T17
	RTelui	*Sle*T17
	GInas	nil
	GBongsu	*Sle*T11
	Belum	nil
	Piah	*Sle*605
	BHijau	nil
	Korbu	nil
	Bubu	nil
B	Behrang	*Sle*T15
	Ampang	nil
	HGombak	*Sle*T29
	HLangat	nil
	SLalang	*Sle*T31
	PPanjang	nil
	Berembun	nil
	Angsi	*Sle*T17
	Kenaboi	nil
	Triang	nil
	Pasoh	nil
	BSenggeh	nil
	GLedang	*Sle*T17, *Sle*465
	Klau	*Sle*T15
	TNegara	nil
	Terenggun	nil
	SBetis	nil
	USat	*Sle*T31
	CTongkat	nil
	HTerengganu	nil
	Jengai	nil
	AGading	nil
	Tekam	nil
	Beserah	nil
	Jengka	*Sle*T17
	Lentang	nil
	Lesong	nil
	ERompin	nil
	GArong	nil
	Labis	nil
	AHitam	nil
	Panti	*Sle*T31

Table S4. Evaluations of deviation from Hardy-Weinberg equilibrium (HWE) for the 10 SSR loci at the population level for *Shorea leprosula*.

Amplified region	Primer name	Primer sequence (5' – 3')	References
*atp*B-*rbc*L	*atp*B	F: CRGGTTGAGGAGTTACTCG	[1]
	*rbc*L	R: ATGTCCCGTTATCGAGGACCT	[1]
*pet*G-*trn*P	*pet*G	F: GGTCTAATTCCTATAACTTTGGC	[2]
	*trn*P	R: GGGATGTGGCGCAGCTTGG	[2]
*trn*L1-*trn*L2	*ucp-c*	F: CGAAATCGGTAGACGCTACG	[3]
	ucp-d	R: GGGGATAGAGGGACTTGAAC	[3]
*psb*M-*trn*D	*psb*M-f2	F:ATAAATGCAAGAATATTTACTTCC	[4]
	*trn*D-M	R: GGGATTGTAGTTCAATTGGT	[5]

Table S5. Four chloroplast DNA primer pairs used in the study.

References

[1] J.D. Chung, T.P. Lin, Y.L. Chen, Y.P. Cheng, S.Y. Hwang, Phylogeographic study reveals the origin and evolutionary history of a Rhododendron species complex in Taiwan, Mol. Phylogenet. Evol. 42(1) (2007) 14-24.
[2] S.-F. Huang, S.-Y. Hwang, J.-C. Wang, T.-P. Lin, Phylogeography of Trochodendron aralioides (Trochodendraceae) in Taiwan and its adjacent areas, J. Biogeogr. 31(8) (2004) 1251-1259.
[3] P. Taberlet, L. Gielly, G. Pautou, J. Bouvet, Universal primers for amplification of three non-coding regions of chloroplast DNA, Plant Mol. Biol. 17 (1991) 1105-1109.
[4] B. Heinze, A database of PCR primers for the chloroplast genomes of higher plants, Plant Methods 3(1) (2007) 1-7.
[5] B. Demesure, N. Sodzi, R.J. Petit, A set of universal primers for amplification of polymorphic non-coding regions of mitochondrial and chloroplast DNA in plants, Mol. Ecol. 4(1) (1995) 129-31.

Locus	Repeat motif	Primer sequence 5'-3'	Allele size range (bp)	Fluorescent label	GenBank accession no
*Sle*T01	$(GCAG)_6$	F: GACCCGATCCAAAGCCATGA	217-254	6-FAM	ON454543
		R: AGTCCACACTGTTAATTCCAGGA			
*Sle*T09	$(GTTA)_{17}$	F: GCCAAAAGCAAGTATGAGGCA	216-297	6-FAM	ON454544
		R: AGTTATTTTGGGATAGAGTAGGTCA			
*Sle*T11	$(GTTG)_6$	F: TGGGACTCCTATGAGCAGCT	265-308	HEX	ON454545
		R: CGCCACCTTGGTCCACATAA			
*Sle*T15	$(AGAC)_6$	F: TGATCGCAAAGGAGCTGTGT	98-121	HEX	ON454546
		R: AATCGTTTCAGAGGCGCAGA			
*Sle*T17	$(TATG)_6$	F: TGCAAGGTTTCTTTAGGCCTG	202-231	HEX	ON454547
		R: TTCCGACACAGATCCCACAA			
*Sle*T29	$(ACCG)_6$	F: GCAGTTGGCTTTCGGTTAGC	151-192	HEX	ON454548
		R: GGAGGCGAACATGGAAGACA			
*Sle*T31	$(GTTT)_6$	F: GACAGCTTGTGGCCCTCTAG	103-151	6-FAM	ON454549
		R: AGTAGTAGCACAGACCTAACGA			
*Sle*267	$(GA)_{17}$	F: CTTAATTGTGATGCCTGTTG	103-141	HEX	AJ616878
		R: TCTTGTATTTATGCTTCTCC			
*Sle*465	$(CT)_{14}$	F: CAAAGTTGAACCTTGAATCT	53-83	HEX	AJ616887
		R: ATTTAGAAGCCGTCCAGC			
*Sle*605	$(GA)_{13}$	F: GTGCATTTATTGCCTGAGTAAGGTGG	115-183	6-FAM	AJ616891
		R: CAACTAAAATGGACCAGACCGGATG			

Table S6. Locus name, repeat motif, primer sequence, allele size range, fluorescent label and GenBank accession number of the 10 simple sequence repeats used for genotyping *Shorea leprosula* in the individual database.

Supplemental material S1

Development of transcriptome-derived SSR markers in *Shorea leprosula*

RNA isolation

Total RNA was extracted from fresh leaf tissue frozen in liquid nitrogen using an RNeasy Plant Mini kit (Qiagen), and subsequently treated with DNase from a Turbo DNA-free kit (Thermo Fisher Scientific, Lithuania) according to the manufacturer's protocol.

Transcriptome sequencing and *de novo* assembly

Illumina's 'TruSeq RNA Sample Prep Kit v2' (Illumina, San Diego, CA, USA) was used to create cDNA libraries according to the manufacturer's protocol. cDNA libraries were then quality-assayed using a Bioanalyzer (Agilent, USA) and sequenced on HiSeq 4000 (Illumina, Hayward, CA, USA). Raw reads were filtered with Trimmomatic v.0.32[1] and cleaned reads were *de novo* assembled using TRINITY v.2.8.4[2]. All assembled transcripts were then used for SSR detection.

Detection of SSR markers

An in-house Shell script was used to mine SSR from the assembled *S. leprosula* contigs using MISA[3] and Primer3 v.4.1.0[4] for primer design. Candidate microsatellites ranged from two to six nucleotides, with the minimum repeat unit defined as six repeats for dinucleotides and five repeats for all higher order motifs. Primer design was carried out with default settings to generate PCR products ranging in size from 100 to 300 bp. We selected SSR loci with a minimum of 10 repeats for dinucleotides, seven repeats for

trinucleotides, five for tetranucleotides and pentanucleotides. Characterization of markers was according to the procedure used for SSR markers delimited in Lee et al. 2004[5].

References

1 Bolger, A. M., Lohse, M. & Usadel, B. Trimmomatic: a flexible trimmer for Illumina sequence data. *Bioinformatics* **30**, 2114-2120, doi:10.1093/bioinformatics/btu170 (2014).

2 Grabherr, M. G. *et al.* Trinity: reconstructing a full-length transcriptome without a genome from RNA-Seq data. *Nat. Biotechnol.* **29**, 644-652, doi:10.1038/nbt.1883 (2011).

3 Thiel, T., Michalek, W., Varshney, R. & Graner, A. Exploiting EST databases for the development and characterization of gene-derived SSR-markers in barley (Hordeum vulgare L.). *Theor. Appl. Genet.* **106**, 411-422 (2003).

4 Rozen, S. & Skaletsky, H. in *Bioinformatics methods and protocols* 365-386 (Springer, 2000).

5 Lee, S., Tani, N., Ng, K. & Tsumura, Y. Isolation and characterization of 20 microsatellite loci for an important tropical tree Shorea leprosula (Dipterocarpaceae) and their applicability to S. parvifolia. *Mol. Ecol. Notes* **4**, 222-225 (2004).
